# Supplementary material for: COVID-19 Social Restrictions’ Impact on the Health-Related Physical Fitness of the Police Cadets
Source: Healthcare (Basel). 2023 Jul 5;11(13):1949. doi: 10.3390/healthcare11131949 (PMC10340710; doi:10.3390/healthcare11131949)
Supplement: Supplementary file 1 [file healthcare-11-01949-s001.zip › healthcare-2430320-supplementary.pdf]

Figure S1. Schematic representation of the ISCPSI slalom test (adapted from ISCPSI protocol)

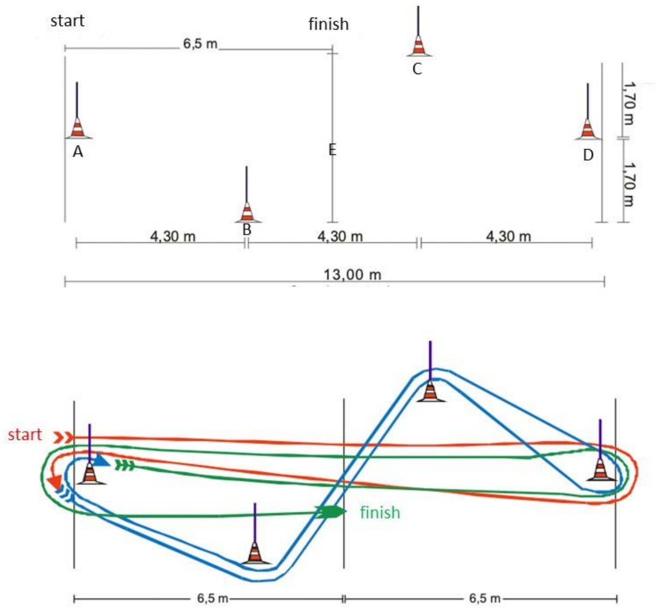

A standard slalom course is made up of four cones (A, B, C and D), placed according to figure 1, and the test (i) begins at A, then a straight line to D, and then back to A; (ii) a standard zig-zag course from A to B, C, D, then back to C, B, A; (iii) then a straight line from A to D, then back to A; and (iv) then a straight line from A to E (finish). The test stopped when the participant: (i) did not start from the stopped position; (ii) did not perform the test course in the correct order; (iii) did not circled the cone (on the outside); or (iv) touched or knocked down any cone.

Table S1. Changes in anthropometric indicators and physical fitness indicators between pre- and post-pandemic social restrictions within sex

	Women (n=46)					Men (n=110)				
**Anthropometric indicators**	Pre Mean±SD	Post Mean±SD	*r*	t	Effect size[d]	Pre Mean±SD	Post Mean±SD	*r*	t	Effect size[d]
Weight (kg)	61.3±7.5	61.1±8.64	**0.950**	0.545	0.08	75.4±8.6	76.4±8.9	**0.921**	**-3.208**	0.31
Body mass index (kg/m^2)	21.9±2.2	21.8±2.7	**0.937**	0.557	0.08	23.8±2.3	24.2±2.4	**0.891**	**-3.246**	0.31
**Physical fitness**										
Speed (sec)	4.8±0.2	4.9±0.3	**0.870**	**-3.209**	0.49	4.2±0.2	4.2±0.2	**0.760**	-1.287	0.12
Agility (sec)	24.7±1.0	25.0±1.3	**0.776**	**-2.204**	0.34	22.5±1.1	22.7±1.0	**0.756**	**-2.855**	0.28
Cardiorespiratory fitness (no. laps)	62.1±13.7	56.0±13.8	**0.649**	**3.458**	0.53	87.7±16.4	77.5±17.5	**0.670**	**7.693**	0.74
$VO_{2\ max}$ (ml/kg/min)	39.9±4.6	37.9±4.7	**0.647**	**3.327**	0.52	48.2±4.9	45.0±5.4	**0.647**	**7.751**	0.75
Lower body strength (m)	2.0±0.2	1.9±0.2	**0.860**	**2.296**	0.35	2.4±0.1	2.4±0.1	**0.742**	0.759	0.07
Flexibility (cm)	56.5±5.7	55.6±5.9	**0.785**	1.520	0.23	52.2±6.6	49.5±7.2	**0.793**	**6.283**	0.61
Static strength (kg[f])	68.4±11.3	62.7±11.9	**0.719**	**4.008**	0.66	104.1±15.6	99.5±14.4	**0.838**	**5.270**	0.54
Abdominal strength (no. reps/60sec)	54.1±6.0	53.5±6.3	**0.345**	0.532	0.08	58.6±6.7	56.5±7.7	**0.574**	**3.128**	0.30

r=Pearson correlation; t=pair-sample t-test; [d]=cohen's d; **bold**=p<0.05 within sex comparisons (pre- and post-pandemic social restrictions).
sec=seconds; cm=centimetres; no.=number; reps=repetitions.

Table S2. Changes in anthropometric indicators and physical fitness indicators between pre- and post-pandemic social restrictions by age.

	18-24 years (n=81)					25-30 years (n=41)					31-36 years (n=34)				
**Anthropometric indicators**	Pre Mean±SD	Post Mean±SD	*r*	t	Effect size[d]	Pre Mean±SD	Post Mean±SD	*r*	t	Effect size[d]	Pre Mean±SD	Post Mean±SD	*r*	t	Effect size[d]
Weight (kg)	67.7±9.8	67.8±10.3	**0.957**	-0.257	0.03	77.0±10.0	77.9±10.6	**0.948**	-1.693	0.27	73.1±9.2	74.9±10.3	**0.931**	**-2.892**	0.50
Body mass index (kg/m^2)	22.4±2.2	22.4±2.4	**0.910**	-0.143	0.02	24.5±2.1	24.8±2.5	**0.905**	-1.789	0.28	23.8±2.4	24.4±2.7	**0.896**	**-2.913**	0.50
**Physical fitness**															
Speed (sec)	4.4±0.3	4.4±0.3	**0.911**	-0.497	0.06	4.3±0.3	4.4±0.4	**0.935**	**-2.443**	0.40	4.4±0.4	4.5±0.4	**0.946**	**-2.577**	0.44
Agility (sec)	23.0±1.3	23.2±1.4	**0.889**	**-2.254**	0.26	22.9±1.6	23.1±1.5	**0.880**	-1.848	0.30	23.4±1.5	23.8±1.7	**0.833**	**-2.110**	0.37
Cardiorespiratory fitness (no. laps)	80.5±21.5	72.0±20.9	**0.802**	**5.663**	0.64	82.4±17.1	72.2±17.3	**0.629**	**4.174**	0.69	77.9±17.1	68.7±16.8	**0.770**	**4.617**	0.79
$VO_{2\ max}$ (ml/kg/min)	46.0±6.6	43.2±6.6	**0.788**	**5.611**	0.64	46.5±5.4	43.3±5.6	**0.640**	**4.167**	0.69	45.1±5.4	42.2±5.4	**0.775**	**4.689**	0.80
Lower body strength (m)	2.3±0.3	2.3±0.3	**0.942**	0.371	0.04	2.3±0.2	2.3±0.3	**0.889**	0.882	0.15	2.3±0.2	2.2±0.3	**0.901**	2.009	0.35
Flexibility (cm)	53.2±6.6	51.1±7.7	**0.810**	**4.059**	0.45	53.7±7.3	51.1±7.7	**0.822**	**3.593**	0.58	53.7±6.1	51.8±6.2	**0.807**	**2.956**	0.51
Static strength (kg^f)	87.7±21.1	82.4±20.3	**0.909**	**3.526**	0.60	103.9±20.6	98.0±21.4	**0.914**	**3.874**	0.67	98.8±19.3	95.7±19.3	**0.918**	**2.062**	0.40
Abdominal strength (no. reps/60sec)	58.7±6.5	56.0±7.5	**0.537**	**5.011**	0.40	55.6±6.7	55.4±7.0	**0.577**	0.264	0.04	55.9±7.0	55.4±7.9	**0.562**	0.421	0.07

r=Pearson correlation; t=pair-sample t-test; [d]=cohen's d; **bold**=$p<0.05$.
sec=seconds; cm=centimetres; no.=number; reps=repetitions
